# Preoperative Neutrophil-Lymphocyte Ratio Can Predict Outcomes for Patients Undergoing Tetralogy of Fallot Repair

**DOI:** 10.21470/1678-9741-2020-0408

**Published:** 2021

**Authors:** Valdano Manuel, Leonardo A. Miana, Gustavo Pampolha Guerreiro, Aida Turquetto, Rômullo Medeiros Santos, Natália Fernandes, Davi Freitas Tenório, Luiz Fernando Caneo, Fabio B. Jatene, Marcelo Biscegli Jatene

**Affiliations:** 1 Division of Cardiovascular Surgery, Instituto do Coração (InCor), Hospital das Clínicas, Faculdade de Medicina, Universidade de São Paulo, São Paulo, São Paulo, Brazil.; 2 Cardio-Thoracic Center, Clínica Girassol, Luanda, Angola.

**Keywords:** Neutrophils, Tetralogy of Fallot, Congenital Heart Disease, Inflammation, Lymphocytes, Biomarkers, Intensive Care Units

## Abstract

**Introduction:**

Elevated neutrophil-lymphocyte ratio (NLR) has been associated with poorer outcomes in cyanotic patients undergoing single ventricle palliation. Little is known about this biomarker on patients with tetralogy of Fallot (TOF), the most common cyanotic congenital heart disease. Our objective is to study the impact of preoperative NLR on outcomes of TOF patients undergoing total repair.

**Methods:**

This retrospective study included 116 consecutive patients between January 2014 and December 2018. Preoperative NLR was measured from the last complete blood count test before the surgery. Using the cutoff value of 0.80, according to the receiver-operating characteristic (ROC) curve, the sample was divided into two groups (NLR < 0.80 and ≥ 0.80). The primary endpoint was hospital length of stay (LOS).

**Results:**

ROC curves showed that higher preoperative NLR was associated with longer hospital LOS, with an area under the curve of 0.801±0.040 (95% confidence interval 0.722 - 0.879; P<0.001). High preoperative NLR was also associated with long intensive care unit (ICU) LOS (P=0.035). Preoperative NLR predicted longer hospital LOS with a sensitivity of 63% and a specificity of 81.4%.

**Conclusion:**

Higher preoperative NLR was associated with long ICU and hospital LOS in patients undergoing TOF repair.

**Table:** 

Abbreviations, acronyms & symbols				
ASD	= Atrial septal defect		MPA	= Main pulmonary artery
CHD	= Congenital heart disease		MV	= Mechanical ventilation
CPB	= Cardiopulmonary bypass		NLR	= Neutrophil-lymphocyte ratio
DS	= Down syndrome		PDA	= Patent ductus arteriosus
ECMO	= Extracorporeal membrane oxygenation		PFO	= Patent foramen ovale
HLOS	= Hospital length of stay		ROC	= Receiver-operating characteristic
ICU	= Intensive care unit		SIRS	= Systemic inflammatory response syndrome
IL	= Interleukins		TNF-α	= Tumor necrosis factor-alpha
IQR	= Interquartile range		TOF	= Tetralogy of Fallot
LOS	= Length of stay		X-clamp	= Cross-clamping
miR	= Micro ribonucleic acid			

## INTRODUCTION

Inflammation occurs in all surgical procedures since it is a physiological response to trauma. However, in major surgeries such as those with cardiopulmonary bypass (CPB), this response may be exacerbated. Exacerbated inflammatory response may cause pathological complications in the postoperative period of congenital heart disease (CHD) repair ^[1-4]^. It causes a deleterious effect in different organs increasing morbidity and mortality in the immediate postoperative period ^[5]^.

Well-known risk factors for exacerbated inflammatory response are longer CPB time ^[5]^ and aortic cross-clamping (X-clamp) times, postoperative liver dysfunction, preoperative leukocytosis, younger age, and low weight ^[5,6]^.

Chronic hypoxemia is directly involved in the pathogenesis of lethal injury to the myocardium, responsible for an imbalance between pro-inflammatory and anti-inflammatory responses in the preoperative period, which is exacerbated after surgical trauma and CPB. Furthermore, neutrophils are just first responders to inflammatory signals released to face the cellular and tissular damage, and the physiological impact of neutrophil interactions with myocardial cells may lead to the preoperative elevation of the neutrophil-lymphocyte ratio (NLR) ^[5-9]^.

Some studies have shown an association between preoperative elevated pro-inflammatory mediators such as interleukins (IL), tumor necrosis factor-alpha (TNF-α), micro ribonucleic acid (miR), peptide amino-terminal procollagen type III, and natriuretic peptides, and increased morbidity and mortality in hypoxemic patients compared to those in acyanotic patients ^[3,7,10-12]^. These specific biomarkers are expensive and are not readily available.

Manuel et al. ^[13]^, Savluk et al. ^[14]^, Xu et al. ^[15]^, and, more recently, Iliopoulos et al. ^[16]^ have, however, presented the prognostic value of the preoperative and postoperative NLR in children undergoing congenital heart surgery.

Little is known about the role of preoperative NLR in predicting outcomes for patients with CHD amenable to complete biventricular repair (*i.e*., tetralogy of Fallot [TOF]). ^[17]^ Calculation of NLR is an inexpensive and widely available exam for all patients undergoing cardiac surgery.

We hypothesized that there is a correlation between elevated preoperative NLR and poorer outcomes in patients undergoing TOF repair. Based on this, we sought to determine whether preoperative NLR might be a predictor of longer hospital length of stay (HLOS) in these patients.

## METHODS

Patients undergoing TOF repair between January 2014 and December 2018 were retrospectively enrolled. The study was conducted at the Instituto do Coração (or InCor) of the Universidade São Paulo, São Paulo, Brazil. The institutional review board and ethics committee approved the study (approval number 1.856.909).

We included all patients who underwent TOF repair and had a complete white blood cell count with differential available preoperatively. All included patients underwent modified ultrafiltration in the surgery.

Exclusion criteria included:

1. Surgery other than TOF repair
2. Previous surgery (Blalock-Taussig shunt included)
3. Association with other procedures (except patent ductus arteriosus ligation and patent foramen ovale [PFO] or atrial septal defect [ASD] closure or pulmonary arteries enlargement) or cardiac anomalies (*e.g*., partial or total atrioventricular septal defect, pulmonary atresia, intact ventricular septum, pulmonary valve agenesis, coronary artery anomaly, severe pulmonary artery stenosis, or any other hemodynamically significant CHD)
4. Preoperative hemodynamic instability
5. Surgical complication leading to higher CPB and X-clamp times
6. Suspected or confirmed infection prior antibiotic administration during the same hospital admission
7. Absence of complete white blood cells count with differential before surgery

Preoperative demographic data included patient’s age, sex, and weight at the time of the surgery, O_2_ saturation, main pulmonary size with z-score, preoperative ventricular function, other associated CHD, presence of chromosomal or structural anomalies, need for preoperative mechanical ventilation (MV), most recent preoperative total neutrophils, and total lymphocytes obtained from the peripheral blood samples before surgery (< 72 hours).

Intraoperative variables included CPB and X-clamp times, need for a transannular patch, and associated procedures. All patients received steroids in the operating room.

Postoperative variables studied included occurrence of ventricular dysfunction, need for extracorporeal membrane oxygenation (ECMO), and significant complications such as cardiopulmonary, neurologic, and infection-related (deep or superficial wound infection) complications, and arrhythmia and readmission. We also analyzed the middle-term survival.

The primary endpoint measured was HLOS. The secondary endpoints were MV time, in-hospital mortality and intensive care unit (ICU) length of stay (LOS), ventricular dysfunction, complications, and readmission.

### Definition of Variables

**NLR** was defined as the ratio of the absolute count of neutrophils to lymphocytes. The patients were divided into groups based on the NLR as follows: Group I (NLR < 0.80) and Group II (NLR ≥ 0.80) according to the receiver-operating characteristic (ROC) curve.

**Longer HLOS** was defined as more than 14 days of hospitalization from the period of surgery until the postoperative discharge. According to the Society of Thoracic Surgeons National Database 2019 Annual Report, the expected LOS for TOF is 12.6 days ^[18]^. We gave it a margin of another day and a half.

**Ventricular dysfunction** was defined as an ejection fraction < 55% on the echocardiogram performed during the postoperative period for the left or right ventricle. In our institution, ventricular function is considered normal when the ejection fraction is > 55%. With aim to evaluate different degrees of dysfunction, we evaluated not only the most severe dysfunction.

**Thirty-day surgical mortality** was defined as death (from any cause) of patients from the present cohort within 30 days postoperatively.

### Statistical Analysis

Standard descriptive statistics were calculated. Continuous numerical variables are presented as median and interquartile range (IQR) (25^th^ - 75^th^ percentiles). ROC curve analysis was used to determine the optimal cutoff levels of the preoperative NLR that predicts HLOS. The Chi-square test and Fisher's exact test were used for categorical variables. Survival was estimated using the Kaplan-Meier curve. The Mann-Whitney U test was used to compare groups. The level of statistical significance was set at *P*<0.05. The data were analyzed using IBM Corp. Released 2015, IBM SPSS Statistics for Windows, Version 23.0, Armonk, NY: IBM Corp. and MedCalc statistical software version 19.1.3.

## RESULTS

### Preoperative Data (Table 1)

A total of 116 patients met the inclusion criteria and were included in the statistical analysis. In Group I (69 patients), the total neutrophil was 2948/mm^3^ (IQR: 2036 - 3694) and the total lymphocytes was 6811/mm^3^ (IQR: 5488 - 9067). The median preoperative NLR was 0.44 (IQR: 0.33 - 0.60). In Group II (47 patients), the total neutrophil was 5060/mm^3^ (IQR: 3713 - 6745) and the total lymphocytes was 3743 (IQR: 2684 - 4435); in this group, the median preoperative NLR was 1.37 (IQR: 1.03 - 1.97). The median age was nine months (IQR: 6 - 13) in Group I and 10 months (IQR: 7 - 26) in Group II (*P*=0.09). Males accounted for 58.6% (68 patients) of the study population. Genetic syndrome or chromosomal abnormality was present in 12.1% of patients and the most common was Trisomy 21 (7.8%). The number of patients with a genetic syndrome was higher in Group II (Table 1). There was no association between the preoperative NLR level and the degree of hypoxia (*P*=0.41) or gender (*P*=0.57).

**Table 1:** Baseline characteristics of 116 patients with tetralogy of Fallot who underwent surgical correction from January 2014 to December 2018.

Variable	Group I (69 patients)	Group II (47 patients)	*P*-value
Gender (male:female)	39:30:00	29:18:00	0.57
Age (months)	9 (IQR: 6 - 13)	10 (IQR: 7- 26)	0.09
Weight (kg)	7.5 (IQR: 6.6 - 8.6)	7.8 (IQR: 5.9 - 10.5)	0.60
O_2_ saturation (%)	92 (IQR: 87 - 95)	90 (IQR: 85 - 95)	0.41
MPA size (mm)	8 (IQR: 6 - 11)	9 (IQR: 6 - 13)	0.19
Z-score for MPA (mm)	-1 (IQR: -1 and -1)	-4 (IQR: -5 and 0)	0.65
Preoperative ventricular dysfunction	1 (1.4%)	0	0.40
Associated diagnosis	23 (33.3%)	19 (40.4%)	0.43
Preoperative mechanic ventilation	2 (2.9%)	4 (8.5%)	0.18
Genetic syndrome	3 (4.3%)	11 (23.4%)	0.002*
Down syndrome	2 (2.9%)	7 (14.9%)	
DiGeorge syndrome	0	2 (4.3%)	
Other	1 (1.4%)	2 (4.3%)	
Total neutrophil	2948/mm^3^ (IQR: 2036 - 3694)	5060/mm^3^ (IQR: 3713 - 6745)	< 0.001
Total lymphocytes	6811/mm^3^ (IQR: 5488 - 9067)	3743/mm^3^ (IQR: 2684 - 4435)	< 0.001
NLR	0.44 (IQR: 0.33 - 0.60)	1.37 (IQR: 1.03 - 1.97)	< 0.001

* Statistically significant values are in bold (*P*<0.05) IQR=interquartile range; MPA=main pulmonary artery; NLR=neutrophil-lymphocyte ratio

### Intraoperative Data (Table 2)

There was no difference in CPB and X-clamp times between groups (Table 2). Minor additional procedures took place in 36.2% of the cases with no statistically significant difference between groups (*P*=0.43).

**Table 2:** Intraoperative data complications and mortality of surgical correction of 116 patients with tetralogy of Fallot from January 2014 to December 2018.

Variables	Group I (69 patients)	Group II (47 patients)	*P*-value
CPB	130 (IQR: 112 - 146)	125 (IQR: 110 - 152)	0.91
Cross-clamping time	103 (IQR: 88 - 116)	97 (IQR: 82 - 117)	0.46
Transannular patch	39 (56.5%)	25 (53.2%)	0.65
Ventricular dysfunction	6 (8.7%)	2 (4.3%)	0.35
Associated procedures	23 (33.3%)	19 (40.4%)	0.43
ASD closure	9 (13%)	11 (23.4%)	
PDA occlusion	4 (5.8%)	2 (4.3%)	
PFO occlusion	7 (10.1%)	2 (4.3%)	
Pulmonary artery enlargement with patch	1 (1.4%)	4 (8.5%)	
ASD and pulmonary repair	2 (2.9%)	0	
Complications	10 (14.5%)	8 (17%)	0.71
Neurologic	3 (4.3%)	2 (4.2%)	
Respiratory	1 (1.4%)	1 (2.1%)	
Infection	1 (1.4%)	3 (6.4%)	
Cardiogenic shock	1 (1.4%)	2 (4.2%)	
Others	4 (5.8%)	0	
Arrhythmia	6 (8.7%)	5 (10.6%)	0.72
Readmission	4 (5.8%)	3 (6.4%)	0.89
In-hospital mortality	1 (1.4%)	4 (8.5%)	0.13

ASD=atrial septal defect; CPB=cardiopulmonary bypass; IQR=interquartile range; PDA=patent ductus arteriosus; PFO=patent foramen ovale Group I neutrophil-lymphocyte ratio (NLR) < 0.80 and Group II NLR ≥ 0.80

### Outcomes

When the data regarding MV time were compared (*P*=0.12), *i.e*., ventricular dysfunction (*P*=0.35), need for postoperative ECMO (*P*=0.79), presence of major complications (*P*=0.71), arrhythmia (*P*=0.72), infection (*P*=0.06), and readmission (*P*=0.89), no statistically significant difference was observed (Table 2).

**Fig. 1:**
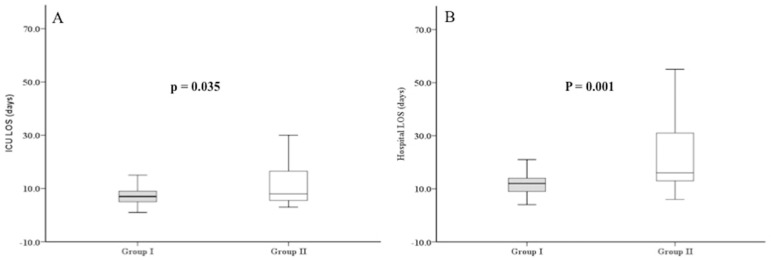
Demonstration of the association of a neutrophil-lymphocyte ratio > 0.80 with prolonged intensive care unit (ICU) length of stay (LOS) (A) and hospital LOS (B).

The median HLOS was 12 days (IQR: 9 - 14) and 16 days (IQR: 15 - 32) for Groups I and II, respectively. The median ICU LOS was seven days (IQR: 5 - 9) and eight days (IQR: 5 - 17) for Groups I and II, respectively (Figure 1).

**Fig. 2:**
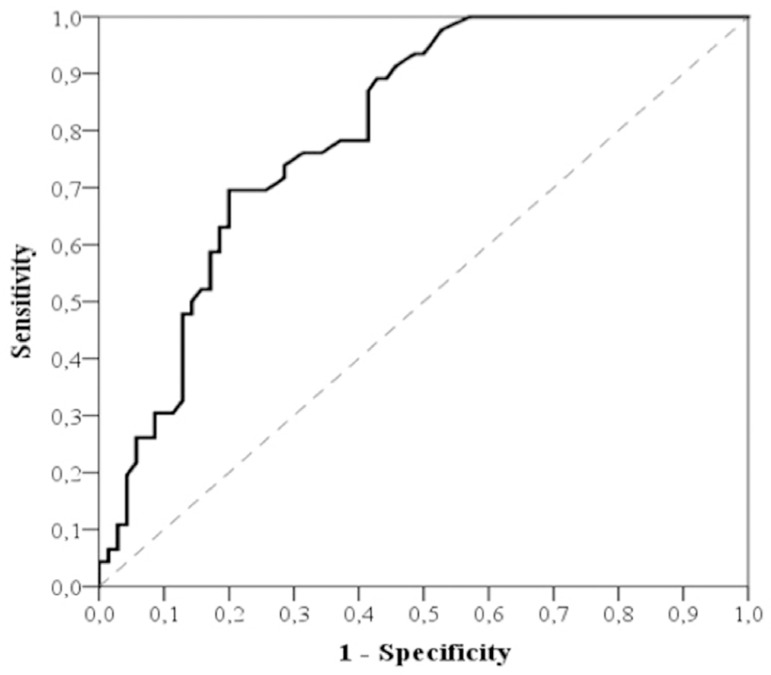
The receiver-operating characteristic analysis of preoperative neutrophil-lymphocyte ratio for postoperative hospital length of stay. The cutoff value was 0.80. The area under the curve was 0.801± 0.040 (95% confidence interval 0.722 - 0.879; P<0.001).

Preoperative NLR was found to be associated with longer HLOS following TOF repair (*P*<0.001), with an area under the ROC curve of 0.801± 0.040 (95% confidence interval 0.722 - 0.879; *P*<0.001). Using a cutoff value of 0.80, the higher preoperative NLR predicted longer postoperative HLOS with a sensitivity of 63% and specificity of 81.4% (Figure 2).

The overall 30-day mortality was 4.3% (five patients). In Group I, one patient expired, and in Group II, four deaths were observed. Causes of death were cardiogenic shock in three patients and sepsis in the two other patients. The Group I patient was a non-syndromic boy with 3.7 months of age and low weight (5.2 kg), saturating 65% in preoperative period with NLR of 0.63; he was not under MV. In Group II, of four patients, one had Down syndrome (DS), two were under preoperative MV, the NLR varied from 0.80 to 5.79, and all of them had low weight and were male.

## DISCUSSION

Our findings revealed that patients undergoing TOF repair with higher preoperative NLR were associated with longer HLOS. Although in-hospital mortality for patients after TOF repair is low, postoperative morbidities may still occur and need to be mitigated ^[19]^. Many of these morbidities are a consequence of an exacerbated inflammatory response to surgical trauma and CPB ^[1-4]^. This physiological inflammatory response has a deleterious effect in one or more organs causing dysfunction on the heart (negative inotropic), lung (respiratory dysfunction), kidney (acute renal dysfunction), liver (liver dysfunction), and vessels (endothelial dysfunction), and may progress to multiple organ dysfunction, thus increasing morbidity and mortality in these patients ^[5,20]^. Some risk factors for exacerbation of this physiological response are well known, including younger age at the time of surgery, longer CPB and aortic X-clamp time, postoperative liver dysfunction, preoperative leukocytosis, and low body weight ^[5,6,21]^.

The impact of inflammation induced by hypoxemia on the myocardium is similar to that of bacteremia, which can be clinically relevant ^[8,9]^. A cyanotic CHD, as TOF, has been identified as an independent risk factor for developing exacerbated systemic inflammatory response syndrome (SIRS) to CPB ^[5-7]^. In the last 20 years, some studies have shown an association between cyanotic CHD and perioperative pro-inflammatory mediators like IL-1, IL-6, IL-7, IL-8, IL-10, IL-12, IL-17, TNF-α, miR, peptide amino-terminal procollagen type III, B-type natriuretic peptide, and N-terminal proB-type natriuretic peptide. This association has been demonstrated before, during, and after CPB in patients with TOF, and is associated with increased morbidity and mortality when compared to acyanotic patients ^[3,5,7,10-12,21]^.

Chronic hypoxia has been shown to play an important role in inflammation. Hypoxia in children with CHD induces the expression of genes associated with apoptosis and remodeling. On the other hand, hypoxia reduces the expression of genes associated with myocardial contractility and function, induces stress to the myocardium, and consequently induces the expression of a variety of genes including cytokines such as IL-6 and TNF-α, leading to worse outcomes ^[7,9,10,21-23]^.

In 2003, another study confirmed the presence of pro-inflammatory cytokines in the myocardium of children with CHD and these concentrations were higher in patients with TOF ^[7]^.

TNF-α was demonstrated to be responsible for acute lung injury in SIRS after CPB leading to increased MV times, ICU LOS, and HLOS ^[21]^ These specific biomarkers are expensive and are not readily available, especially to patients in the developing world.

Accordingly, Manuel et al. ^[13]^, Savluk et al. ^[14]^, Xu et al. ^[15]^, and, more recently, Iliopoulos et al. ^[16]^ have reported the prognostic value of the preoperative and postoperative NLR in children undergoing congenital heart surgery. NLR is inexpensive and widely available for patients undergoing surgery worldwide. Although there is no study showing correlation between the NLR and cytokines, its advantage is the fact that it is both inexpensive and readily accessible to clinicians worldwide. To the best of our knowledge, an analysis of preoperative NLR as a reliable outcome predictor in patients undergoing TOF repair with positive result has not been done ^[18]^.

Herein, NLR was found to have 81.4% specificity and 63% sensitivity in predicting a longer HLOS. These findings are similar to those described in studies using more complex biomarkers, such as cytokines or miR, as predictors ^[3,5,7,10-12,21]^. Although the sensitivity was low, it is inexpensive and may be helpful for predicting outcome.

The reasons for imbalanced preoperative NLR in hypoxemic patients remain unclear. In previous studies, patients with O_2_ saturation < 90% were found to have higher preoperative or intramyocardial cytokines ^[7,21]^. Although, in the present cohort, the association between hypoxia and inflammation was not statistically significant, patients of Group II were more cyanotic. This may have occurred due to the characteristics of our cohort - many patients had a patent ductus arteriosus, ASD, or PFO - or the cutoff value of O_2_ saturation should be < 90%.

Chronic hypoxia can justify this imbalance because some subtypes of leukocytes such as neutrophils are evolved during the inflammatory response ^[9,20]^. Previous studies have shown an association between elevated cytokine (IL-6) levels and high neutrophil and low lymphocyte in other chronically hypoxemic patients ^[8,9,24,25]^. The stress caused by hypoxia in the heart induces myocardium release of cytokines like IL-6, which is a neutrophil-derived mediator of injury ^[8,9]^. In our cohort, the total neutrophil count was higher, and the total lymphocyte count was lower in patients of Group II (P<0.001).

This imbalance between neutrophil and lymphocytes causing myocardial damage has been shown in association with neutrophilic infiltration causing direct damage after myocardial hypoxia in adult patients undergoing CABG ^[26]^. The inflammatory response to chronic hypoxemia may be similar ^[20]^. It is also implicated in reperfusion injury through direct toxic effects of oxygen-derived free radicals inducing myocardial and endothelial remodeling, which affects blood flow and causes damage to the heart ^[26,27]^.

The number of patients with a genetic syndrome was higher in Group II (mainly DS and DiGeorge syndrome). Children with DS have been associated with altered serum pro-inflammatory cytokines such as IL-5, IL-10, IL-13, and TNF-α; however, a correlation with NLR has not been proven ^[27,28]^ and it is unknown if this had an influence in the observed longer LOS in our study, maybe a subanalysis should be performed. Lal et al. ^[29]^ demonstrated that DS did not affect mortality or ICU LOS after atrioventricular septal defect repair.

CPB itself is associated with neutrophil activation, which may accentuate the effects of high preoperative levels as seen in our patients. CPB is an independent cause of SIRS. Blood contact with non-endothelialized surface induces complement activation, causes the release of reactive oxygen species, arachidonic acid metabolites, and numerous cytokines, and activates leukocytes ^[25,26,30]^. High preoperative NLR (inflammation) may be related to exacerbated postoperative inflammation and, consequently, increased MV time, as well as longer ICU LOS and HLOS, as demonstrated in the present and previous studies ^[3,5,7,10-15,21,23]^.

The data presented here suggest an imbalance between pro-inflammatory and anti-inflammatory responses due to chronic hypoxemia in the preoperative period, which is exacerbated after surgical trauma and CPB. This impacts the outcomes in children with cyanotic CHD.

We observed an expected mortality rate in Group I patients (1.45%), but a higher mortality (although not statistically significant) in Group II (8.5%). Future studies with a larger number of patients are needed to clarify whether a higher NLR impacts in-hospital mortality.

This study provides practical information for specialists in the field since the value of preoperative NLR can guide when TOF repair can be done to maximize outcomes.

### Limitations

One of the main limitations of our study is that it is a single-center, retrospective study. The other is a small number of participants due to the intention to homogenize the sample with the aim of eliminating possible biases.

## CONCLUSION

Elevated NLR ratio was associated with longer ICU LOS and HLOS. The current study does not show any significant changes in the MV or changes suggestive of low cardiac output syndrome. Although the low sensitivity of the preoperative NLR should be considered, it is an inexpensive, universally available test that may be helpful in predicting outcome. Further investigation of this modality is needed to determine its usefulness and reliability. If an association and a correlation between cytokine levels and NLR prove to be reliable, it might be possible to impact outcome if mechanisms to regulate the inflammatory response are employed.

**Table:** 

Authors' roles & responsibilities	
VM	Substantial contributions to the conception and design of the work; and the acquisition and analysis of the data for the work; drafting the work; final approval of the version to be published
LAM	Substantial contributions to the conception and design of the work; drafting the work; final approval of the version to be published
GPG	Substantial contributions to the acquisition of data for the work; final approval of the version to be published
AT	Substantial contributions to the analysis of data for the work; final approval of the version to be published
RMS	Substantial contributions to the acquisition of data for the work; final approval of the version to be published
NF	Substantial contributions to the acquisition of data for the work; final approval of the version to be published
DFT	Substantial contributions to the acquisition of data for the work; final approval of the version to be published
LFC	Revising the work; final approval of the version to be published
FBJ	Revising the work; final approval of the version to be published
MBJ	Revising the work; final approval of the version to be published

## References

[1] Seghaye MC, Duchateau J, Grabitz RG, Nitsch G, Marcus C, Messmer BJ (1994). Complement, leukocytes, and leukocyte elastase in full-term neonates undergoing cardiac operation. J Thorac Cardiovasc Surg.

[2] Soares LC, Ribas D, Spring R, Silva JM, Miyague NI (2010). Perfil clínico da resposta inflamatória sistêmica após cirurgia cardíaca pediátrica com circulação extracorpórea. Arq Bras Cardiol.

[3] Allan CK, Newburger JW, McGrath E, Elder J, Psoinos C, Laussen PC (2010). The relationship between inflammatory activation and clinical outcome after infant cardiopulmonary bypass. Anesth Analg.

[4] Levy JH, Tanaka KA (2003). Inflammatory response to cardiopulmonary bypass. Ann Thorac Surg.

[5] Güvener M, Korun O, Demirtürk OS (2015). Risk factors for systemic inflammatory response after congenital cardiac surgery. J Card Surg.

[6] Bhatia M, Moochhala S (2004). Role of inflammatory mediators in the pathophysiology of acute respiratory distress syndrome. J Pathol.

[7] Qing M, Schumacher K, Heise R, Woltje M, Vazquez-Jimenez JF, Richter T (2003). Intramyocardial synthesis of pro- and anti-inflammatory cytokines in infants with congenital cardiac defects. J Am Coll Cardiol.

[8] Eltzschig HK, Carmeliet P (2011). Hypoxia and inflammation. N Engl J Med.

[9] Vinten-Johansen J (2004). Involvement of neutrophils in the pathogenesis of lethal myocardial reperfusion injury. Cardiovasc Res.

[10] Nagy O, Baráth S, Ujfalusi A (2019). The role of microRNAs in congenital heart disease. EJIFCC.

[11] Zloto K, Tirosh-Wagner T, Bolkier Y, Bar-Yosef O, Vardi A, Mishali D (2018). MiRNA-208a as a sensitive early biomarker for the postoperative course following congenital heart defect surgery. Pediatr Cardiol.

[12] Sugimoto M, Kuwata S, Kurishima C, Kim JH, Iwamoto Y, Senzaki H (2015). Cardiac biomarkers in children with congenital heart disease. World J Pediatr.

[13] Manuel V, Miana LA, Guerreiro GP, Tenório DF, Turquetto A, Penha JG (2020). Prognostic value of the preoperative neutrophil-lymphocyte ratio in patients undergoing the bidirectional glenn procedure. J Card Surg.

[14] Savluk OF, Guzelmeric F, Yavuz Y, Ukil F, Yilmaz A, Cevirme D (2019). Neutrophil-lymphocyte ratio as a mortality predictor for norwood stage I operations. Gen Thorac Cardiovasc Surg.

[15] Xu H, Sun Y, Zhang S (2019). The relationship between neutrophil to lymphocyte ratio and clinical outcome in pediatric patients after cardiopulmonary bypass surgery a retrospective study. Front Pediatr.

[16] Iliopoulos I, Alder MN, Cooper DS, Villarreal EG, Loomba R, Sahay RD (2020). Pre-operative neutrophil-lymphocyte ratio predicts low cardiac output in children after cardiac surgery. Cardiol Young.

[17] Sisli E, Yalçinbas YK, Türkekul Y, Yüksek A, Saygili A, Sarioglu T (2016). Does preoperative neutrophil-lymphocyte ratio indicate postoperative morbidity after repair of tetralogy of Fallot. Turk Gogus Kalp Dama.

[18] Fernandez FG, Shahian DM, Kormos R, Jacobs JP, D'Agostino RS, Mayer JE Jr (2019). The society of thoracic surgeons national database 2019 annual report. Ann Thorac Surg.

[19] Romeo JLR, Etnel JRG, Takkenberg JJM, Roos-Hesselink JW, Helbing WA, van de Woestijne P (2020). Outcomes after surgical repair of tetralogy of Fallot a systematic review and meta-analysis. J Thorac Cardiovasc Surg.

[20] Seghaye MC (2003). The clinical implications of the systemic inflammatory reaction related to cardiac operations in children. Cardiol Young.

[21] Sethi BS, Kapoor PM, Chauhan S, Chowdhury UK, Kiran U, Choudhury M (2014). Perioperative levels of tumor necrosis factor-a correlate with outcomes in children and adults with tetralogy of Fallot undergoing corrective surgery. World J Pediatr Congenit Heart Surg.

[22] Ghorbel MT, Cherif M, Jenkins E, Mokhtari A, Kenny D, Angelini GD (2010). Transcriptomic analysis of patients with tetralogy of Fallot reveals the effect of chronic hypoxia on myocardial gene expression. J Thorac Cardiovasc Surg.

[23] Hövels-Gürich HH, Schumacher K, Vazquez-Jimenez JF, Qing M, Hüffmeier U, Buding B (2002). Cytokine balance in infants undergoing cardiac operation. Ann Thorac Surg.

[24] Jones SM, McCracken C, Alsoufi B, Mahle WT, Oster ME (2018). Association of preoperative cell counts with outcomes after operation for congenital heart disease. Ann Thorac Surg.

[25] Cabrera AG, Dyamenahalli U, Gossett J, Prodhan P, Morrow WR, Imamura M (2009). Preoperative lymphopenia is a predictor of postoperative adverse outcomes in children with congenital heart disease. J Thorac Cardiovasc Surg.

[26] Gibson PH, Croal BL, Cuthbertson BH, Small GR, Ifezulike AI, Gibson G (2007). Preoperative neutrophil-lymphocyte ratio and outcome from coronary artery bypass grafting. Am Heart J.

[27] Wan S, LeClerc JL, Vincent JL (1997). Inflammatory response to cardiopulmonary bypass mechanisms involved and possible therapeutic strategies. Chest.

[28] Nateghi Rostami M, Douraghi M, Miramin Mohammadi A, Nikmanesh B (2012). Altered serum pro-inflammatory cytokines in children with Down's syndrome. Eur Cytokine Netw.

[29] Lal PS, Chavan B, Devendran VR, Varghese R, Murmu UC, Kumar RS (2013). Surgical outcome of congenital heart disease in Down's syndrome. Asian Cardiovasc Thorac Ann.

[30] Huber JN, Hilkin BM, Hook JS, Brophy PD, Davenport TL, Davis JE (2017). Neutrophil phenotype correlates with postoperative inflammatory outcomes in infants undergoing cardiopulmonary bypass. Pediatr Crit Care Med.

